# Honeybee Associative Learning Performance and Metabolic Stress Resilience Are Positively Associated

**DOI:** 10.1371/journal.pone.0009740

**Published:** 2010-03-17

**Authors:** Gro V. Amdam, Erin Fennern, Nicholas Baker, Brenda Rascón

**Affiliations:** 1 School of Life Sciences, Arizona State University, Tempe, Arizona, United States of America; 2 Department of Chemistry, Biotechnology and Food Science, Norwegian University of Life Sciences, Aas, Norway; L'université Pierre et Marie Curie, France

## Abstract

**Background:**

Social-environmental influences can affect animal cognition and health. Also, human socio-economic status is a covariate factor connecting psychometric test-performance (a measure of cognitive ability), educational achievement, lifetime health, and survival. The complimentary hypothesis, that mechanisms in physiology can explain some covariance between the same traits, is disputed. Possible mechanisms involve metabolic biology affecting integrity and stability of physiological systems during development and ageing. Knowledge of these relationships is incomplete, and underlying processes are challenging to reveal in people. Model animals, however, can provide insights into connections between metabolic biology and physiological stability that may aid efforts to reduce human health and longevity disparities.

**Results:**

We document a positive correlation between a measure of associative learning performance and the metabolic stress resilience of honeybees. This relationship is independent of social factors, and may provide basic insights into how central nervous system (CNS) function and metabolic biology can be associated. Controlling for social environment, age, and learning motivation in each bee, we establish that learning in Pavlovian conditioning to an odour is positively correlated with individual survival time in hyperoxia. Hyperoxia induces oxidative metabolic damage, and provides a measure of metabolic stress resistance that is often related to overall lifespan in laboratory animals. The positive relationship between Pavlovian learning ability and stress resilience in the bee is not equally established in other model organisms so far, and contrasts with a genetic cost of improved associative learning found in *Drosophila melanogaster*.

**Conclusions:**

Similarities in the performances of different animals need not reflect common functional principles. A correlation of honeybee Pavlovian learning and metabolic stress resilience, thereby, is not evidence of a shared biology that will give insight about systems integrity in people. Yet, the means to resolve difficult research questions often come from findings in distant areas of science while the model systems that turn out to be valuable are sometimes the least predictable. Our results add to recent findings indicating that honeybees can become instrumental to understanding how metabolic biology influences life outcomes.

## Introduction

Childhood psychometric (IQ) scores correlate with age at death [1]–[3] and can, statistically, predict mortality with a strength similar to that of smoking [4]. Covariance of psychometric scores and longevity is explained by complex inter-related factors, such as socio-economic status, education, health behaviour, disease factors and illnesses, as well as pre- and postnatal privations [2], [3], [5], [6]. Yet, IQ-longevity relationships can remain largely intact when markers of fetal development (birth weight) and early-life conditions (parental social status) are taken into account during statistical processing of data [2], [4]. Such patterns of persistence led to the debated claim (e.g. [3], [7], [8]) that a fraction of covariance in cognition-survival correlations is explained by physiological ‘systems integrity’, a poorly understood factor [2], [5], [9].

Systems integrity encompasses functional reserve capacity and metabolic robustness [4], [9], [10]. The former refers to the capacity to maintain brain function during degenerative processes. The latter to the ability to maintain metabolic stability despite induced oxidative damage. Mechanisms of longevity, and the physiology of central nervous system (CNS) function, ageing, and frailty, are much-studied in genetic workhorses *Caenorhabditis elegans*, *Drosophila*, and mice, where some mutants maintain youthful levels of CNS function at advanced ages [11]–[13]. However, positive correlations between early-life performance of CNS computational processes, such as learning, and physiological stability or survival are generally not measured in prior studies (reviewed by Burger and coworkers [13], see also citations [14]–[16]). In *Drosophila*, furthermore, the strongest correlated response to artificial selection for improved associative learning is shorter lifespan — revealing a negative genetic link between learning ability and survival [13].

Research on poorly understood factors that potentially influence lifespan may ultimately benefit efforts to reduce health and longevity disparities between people [17]–[19]. However, studies motivated by IQ-longevity relationships are debated and difficult to justify. At the same time, it is uncertain whether variables related to early-life CNS computational task performance, such as learning, are positively correlated with survival in the laboratory, and whether these connections can be generalized to model animals. Here, we directly address the latter questions by studying a relationship between a measure of associative learning performance and metabolic stress resilience in the honeybee (*Apis mellifera*).

Social effects have strong influences on honeybee life outcomes [20]–[24]. Individuals that are largely identical genetically can be very different phenotypically, as exemplified by the reproductive division of labour between sister queens (primary egg-layers) and workers (essentially sterile female helpers), and in the social division of labour between workers that move between behavioural roles: nursing, nest building, guarding, colony defence, and foraging [20]. CNS function differs between workers, as measured in laboratory learning and memory retention tests (see citations [25], [26] for recent reviews). In such tests, the individual bee learns to respond to stimuli (olfactory, tactile, visual), and shows different memory forms [27]–[30]. Worker longevity also varies greatly, from weeks to months, and is partly contingent on social role as nurse bees can generally outlive foragers—in the colony as well as in laboratory confinement (reviewed by Amdam and co-workers [31], [32]). Such differences in worker survival correlate with the bees' resistance to laboratory-induced oxidative stress, a test of metabolic stress resilience that nurse bees can endure longer than foragers [32]–[34].

The opportunity to quantify these variables in honeybees led us to examine whether Pavlovian learning ability can be positively correlated with survival during oxidative insult.

## Results and Discussion

We obtained adult worker bees from single-cohort colonies (N = 4), a method that provides animals of known (same) age and social role (see Materials and Methods). To control for social role, we chose a single well-defined behavioural group — nurse bees (young caregivers)—and quantified individual associative learning performance using a well-established procedure for Pavlovian olfactory learning [35]. Nurse bees were trained to a conditioned stimulus (CS) — an odour — which was associated with a sucrose reward (unconditioned stimulus, US). Gustatory responsiveness was determined prior to training as a control for individual motivational state; this responsiveness conveyed the subjective value each animal placed on the US, the sucrose reward [29]. Learning ability was scored on an integer scale from 0 (poorest score) to 5 (best score). Thereafter, individual metabolic stress resilience was measured as survival time in 80% O_2_ (hyperoxia). Hyperoxia induces oxidative stress, metabolic damage, and features of premature senescence in model animals [36]–[38]. This reproducible approach gives a measure of metabolic stress resistance, a variable that often is related to lifespan of model organisms [36], [39], as shown in honeybees [32]–[34].

### Pavlovian learning ability and metabolic stress resilience

**Figure 1:**
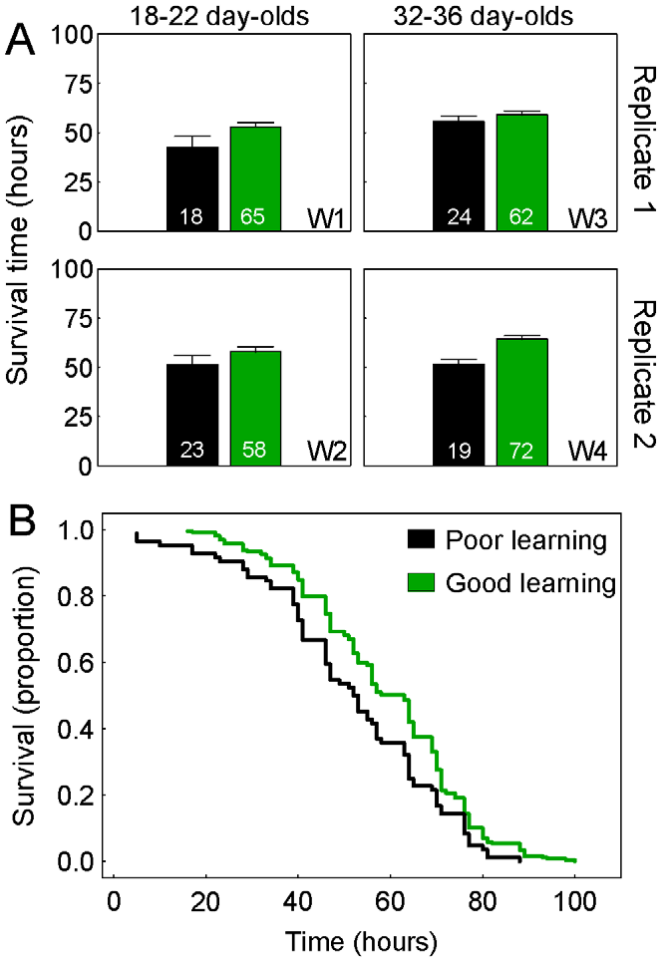
A positive association between Pavlovian learning ability and survival time in worker honeybees. (**A**) Average + S.E. survival time (h) in hyperoxia (80% O_2_) of honeybees with poor (black bars) vs. good (green bars) associative learning ability. Bees were collected in equal numbers from four single-cohort colonies assembled from <24 h old bees (see Materials and Methods). The four colonies were prepared as two pairs, independent Replicate 1 and 2, which were set up one week apart. During the course of the experiment, each replicate pair was tested twice; when bees were 18–22 day-olds (from Replicate 1 during sample week 1 (W1) and from Replicate 2 during W2), and when bees were 32–36 day-olds (from Replicate 1 during W3 and from Replicate 2 during W4). In hyperoxia, the survival time of workers with poor performance (learning score 0–1) was shortened compared with the bees that had performed better in Pavlovian learning (scores 4–5). Sample sizes inside bars. (**B**) Proportional survival probability during the time course of metabolic insult in hyperoxia, summing over the workers shown in panel A (N = 341). Learning ability and metabolic stress resistance are positively connected. Compared to the individuals with poor learning scores (N = 84), bees that did well in associative learning (N = 257) showed significantly higher proportional survival (greater metabolic stress resistance) throughout the experiment.

By comparing all animals with data on learning ability (learning categories 0–5) and subsequent survival time in hyperoxia (between 4–100 h, N = 390), we found a modest but significant positive correlation between individual associative learning performance and longevity (Pearson's correlation; R = 0.11, P = 0.036, N = 390). This pattern was consistent throughout the experiment, and repeatable between independent replicate setups (visualized as mean plots of survival times, Figure 1A). Accordingly, poor learning ability would be a predictor of short survival time in hyperoxia, while good learning performance would be associated with higher resilience and extended survival. We tested the robustness of this connection by excluding bees with mid performance scores in learning (N = 49, learning categories 2–3), thereby strictly comparing workers with the poorest and best performance scores (learning categories 0, 1, 4, and 5). The correlation remained significant (R = 0.15, P = 0.007, N = 341). Next, we used proportional hazard statistics to contrast the survival data from the poorest learners (scores 0–1) toward the bees with the best performance (scores 4–5). This analysis confirmed that associative learning ability was a significant predictor of longevity during laboratory-induced metabolic stress in hyperoxia (Cox's Regression; χ^2^ = 7.259, P = 0.007, N = 341; Fig. 1B).

By using poor vs. good learning in Pavlovian conditioning to an odour (learning categories 0–1 vs. 4–5) as the predictor of survival time in hyperoxia, we could establish that the relationship between honeybee learning ability and metabolic stress resistance persisted when variance from social environment (colony) and age at testing were controlled for (MANOVA; F = 7.03, P = 0.008, N = 341). This analysis showed that the social environment did not influence the bees' longevity in hyperoxia (F = 2.09, P = 0.102), while their age at testing had a positive effect on survival that was independent of learning performance (F = 13.00, P = 0.0004, see also Figure 1A). A comparable response was identified by Seehuus and co-workers [33], who measured increased oxidative stress resistance in mid-aged nurse bees compared with younger bees. Similarly, we used nurse bees in our experiment (Materials and Methods). Seehuus and co-workers attributed the effect of nurse bees' age to vitellogenin, a multifunctional antioxidant protein that can accumulate over time in nurse bees [23], [31], [40], [41]. This physiological factor may also explain the effect of age in our study.

**Figure 2:**
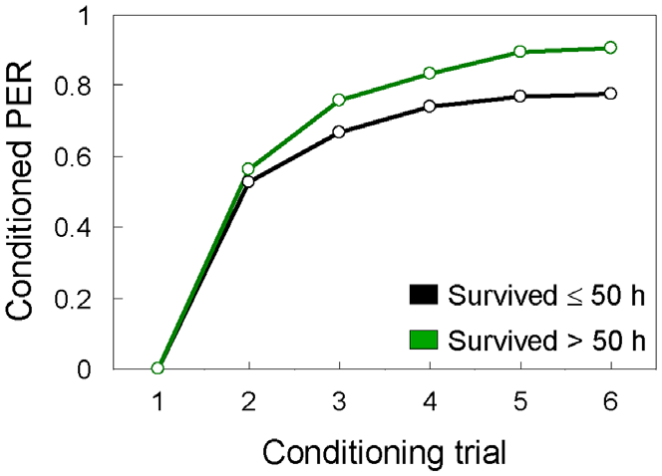
Olfactory learning in worker honeybees with different metabolic stress resilience. Acquisition (learning) curves for the proportion of worker bees that showed conditioned responses to an odour (CS) in each of six conditioning trials. Learning was quantified by the bees' proboscis extension response (PER), which was monitored during every presentation of the odour. Bees that after the conditioning experiment survived ≤50 h (black line, N = 135) vs. >50 h (green line, N = 255) in hyperoxia (80% O_2_) are graphed separately. See text for details on statistics.

Finally, we went back to the full dataset (N = 390) to test whether the positive association between Pavlovian learning ability and subsequent survival time in hyperoxia also influenced the olfactory acquisition (learning) curves of the worker bees. We contrasted the workers that died during the first half of the survival experiment (≤50 h in hyperoxia, N = 135) to bees that died during the second half (>50 h in hyperoxia, N = 255). Plotting the two curves revealed that the increase in conditioned responses was steepest after the initial conditioning trial and then gradually levelled out for both groups (Figure 2). After the second trial, however, the learning curve increased significantly more steeply for workers that survived >50 h in hyperoxia (ANCOVA, one sided test; F = 4.41, P = 0.038, N = 390); and this group also reached higher plateau levels of acquisition (89% in the 6^th^ and final trial) in comparison to those surviving ≤50 h in hyperoxia (77%, Figure 2). These results suggest that faster learning after the initial conditioning trial and a higher level of final memory acquisition characterised the workers with the highest resistance to metabolic stress.

### Gustatory responsiveness and metabolic stress resilience

**Figure 3:**
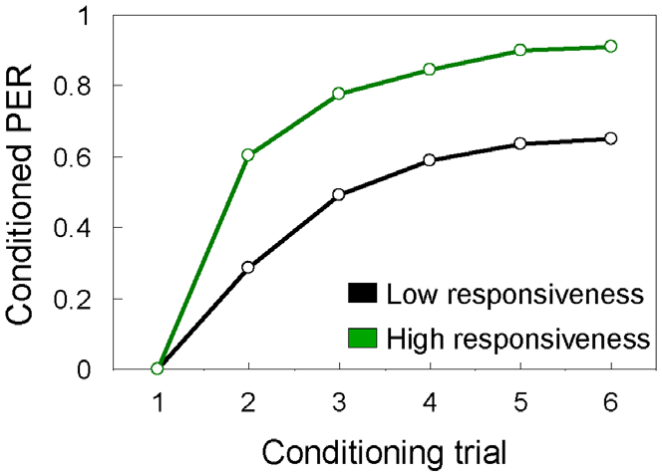
Olfactory learning in worker honeybees with different gustatory responsiveness. Acquisition (learning) curves for the proportion of bees that showed conditioned proboscis extension response (PER) to an odour in six conditioning trials. Bees with different responsiveness to sucrose are graphed separately. Low responsiveness (black line, N = 63) refers to worker bees that did not respond to sucrose at ≤0.1% in H_2_O; High responsiveness (green line, N = 327) refers to bees that did respond to sucrose at ≤0.1% in H_2_O. See text for details on statistics.

Our control data on individual responsiveness to sucrose identified a positive correlation between the gustatory responsiveness score and learning score of the bees. This association was significant in the full dataset (Pearson's correlation; R = 0.33, P<0.001, N = 390) as well as when the workers with the mid performance scores (learning categories 2–3) were excluded (Pearson's correlation; R = 0.36, P<0.001, N = 341). This result corroborated a general finding: bees that place a high subjective value on sucrose rewards often perform better in reward learning [29], [42]. The same result was conveyed by plotting the learning curves of bees with lower gustatory responsiveness (did not respond to sucrose at ≤0.1% in H_2_O, N = 63) toward those with higher gustatory responsiveness (did respond to sucrose at ≤0.1 in H_2_O, N = 327). From the first conditioning trial, the acquisition curve increased significantly more steeply for bees with higher gustatory responsiveness (ANCOVA, one sided test; F = 56.14, P<0.001, N = 390, Figure 3). Thus, a larger percentage of these workers (91%) showed the conditioned response in the final trial compared to the group with lower gustatory responsiveness (0.65%, Figure 3). Faster learning and a higher level of final memory acquisition, accordingly, characterised the bees with higher gustatory responsiveness.

**Figure 4:**
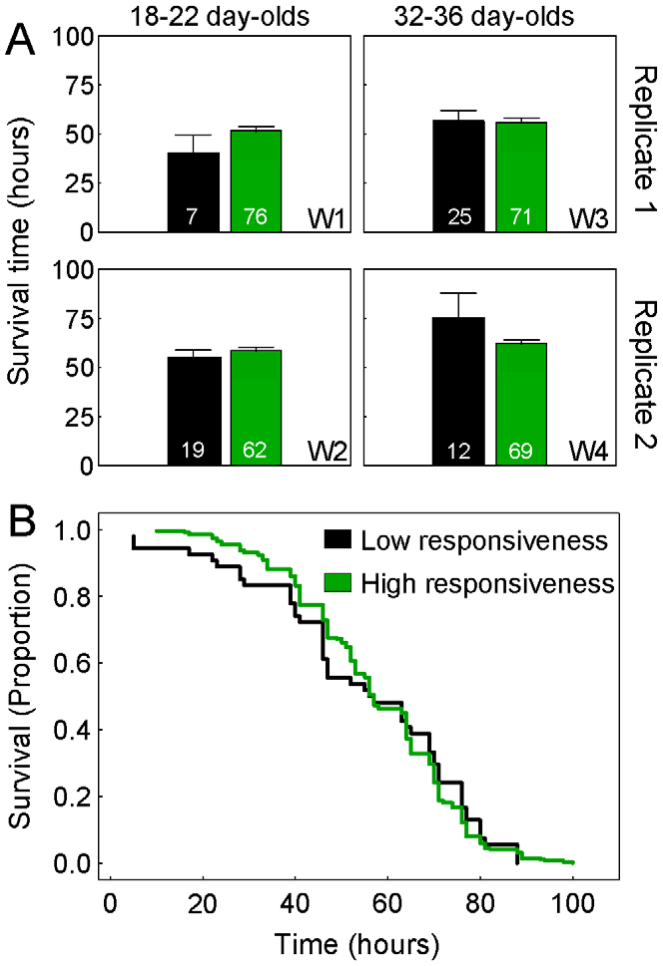
The gustatory responsiveness of honeybee workers is not associated with metabolic stress resistance. (**A**) Average + S.E. survival time (h) in hyperoxia (80% O_2_) of bees with low (black bars) vs. high (green bars) gustatory responsiveness (not responding vs. responding to sucrose at ≤0.1% in H_2_O, respectively). Bees were collected from four single-cohort colonies that were prepared as two pairs one week apart (Replicate 1 and 2). Each replicate pair was tested when bees were 18–22 day-olds (from Replicate 1 during sample week 1 (W1) and from Replicate 2 during W2), and when bees were 32–36 day-olds (from Replicate 1 during W3 and from Replicate 2 during W4). Gustatory responsiveness failed to show a consistent relationship to the bees' subsequent survival time in hyperoxia. Sample sizes inside bars. (**B**) Proportional survival probability during the time course of metabolic insult in hyperoxia, summing over the workers shown in panel A (N = 341). Gustatory responsiveness and metabolic stress resistance are not associated. See text for statistics.

Although responsiveness to sucrose was a predictor of learning performance, and learning performance was a predictor of survival during induced metabolic damage, the bees' appraisal of sucrose rewards did not similarly explain longevity in hyperoxia. This lack of association was seen in the full dataset (R = 0.070, P = 0.167, N = 390) as well as when the workers with mid performance in learning (scores 2–3) were excluded (R = 0.058, P = 0.285, N = 341). The pattern was consistent between our replicate setups (Figure 4A). We also used proportional hazard statistics to contrast bees with lower vs. higher gustatory responsiveness (did not vs. did respond to sucrose at ≤0.1% in H_2_O, respectively). The analysis confirmed that sucrose responsiveness did not predict survival time in hyperoxia (Cox's Regression; χ^2^ = 1.001, P = 0.464, N = 341; Figure 4B).

From these results, we inferred that the variance in learning ability that correlates with metabolic stress resistance in worker bees is independent of the variance that is explained by the bees' subjective motivation to learn. In other words, only a fraction of variation in learning is explained by gustatory responsiveness [29]. Here, this proportion of explained variance, R^2^, was 10.89% (R = 0.33; N = 390, above), which leaves much variation in learning to be explained by factors other than sucrose responsiveness. Our results suggest that one or more of these latent factors, which affect learning but not motivation, can influence metabolic stress resilience — causing learning scores and survival times to correlate independent of the gustatory responsiveness of the bees.

### Conclusions

Our work establishes that in young caregiver (nurse) honeybees, individual performance in Pavlovian olfactory learning is positively associated with metabolic stress resistance measured in hyperoxia. This finding exemplifies that a positive correlation between early-life CNS function and a variable related to organismal survival can be detected in, and perhaps generalized to, a laboratory animal.

While the correlation between learning in Pavlovian conditioning to an odour and subsequent survival time in hyperoxia was modest in our worker bees (Pearson's correlation: R = 11 for the full dataset; R = 15 with mid performance values excluded, above), a Pearson's analysis of correlation between childhood IQ and age at death, similarly, gave only R = 0.18 for 722 human subjects [2]. Thus, our results are statistically significant and in line with the interpretation that positive associations between variables related to CNS computational task-performance (in our case associative olfactory learning) and longevity are moderate.

Bees have rich and quantifiable learning and memory repertoires [25], [26], are amenable to functional genomic research, and provide the best-studied social invertebrate system to date [32], [43]. In this model, genotype, social environment, social history, behaviour, workload, nutrition, physiology, and health can be controlled [32], [43]–[45]—helping us identify and understand mechanisms that affect life-history. Such experiments already propose that life outcomes in social insects can be strongly influenced by metabolic biology [46], [47].

Similarities in patterns of test performance between different organisms need not reflect common functional principles [48], yet it is also difficult to predict which models will become the most valuable for addressing and understanding unresolved challenges in research [49]. Many more studies will need to be conducted before we fully grasp how honeybees can best contribute toward research efforts to reduce health and longevity disparities between people.

## Materials and Methods

### Bees

The experiments were performed in Spring 2009 at Arizona State University in Tempe AZ, USA, and utilized four single-cohort colonies [50], [51]. Each single-cohort colony was assembled with one egg-laying queen and several thousand workers. Within every colony, all workers belonged to one age-cohort. This demography was achieved by collecting honeybee combs with mature brood from a set of nine donor colonies. The combs were placed in an incubator overnight at 33°C in a relative humidity (RH) of 65–70%. The next morning, newly emerged bees (0–24 h old) were collected from the incubator and marked on the thorax with paint (Testors™) for identification.

Two genetic sources were donors of newly emerged bees: *i)* genetically diverse wild type stocks from four colonies headed by openly mated queens of Californian commercial origin, and *ii)* a standard research stock maintained by instrumental insemination, using five colonies headed by queens inseminated with 1–2 drones (males) each. The wild type provided a background population for the single-cohort colonies, but all sampled bees came from the standard research stock, which has a well-documented and broad distribution of learning behaviour [52], [53].

The four colonies were prepared as two separate pairs for independent replication of our experiment. The first paired colonies (Replicate 1) each contained 2,700 wild type workers plus 2,300 bees of standard stock. The second paired colonies (Replicate 2) were assembled with 3,400 wild type workers plus 3,000 bees of standard stock.

### Sampling and handling

For experimental Replicate 1, collections were performed in calendar week 20 (bees aged 18–22 days old) and 22 (bees aged 32–36 days old). Sampling for Replicate 2 took place during calendar weeks 21 (18–22 day-olds) and 23 (32–36 day-olds). These staggered collections provided two replicates of age-matched bees. Collections started at 7 AM, and only marked bees of the standard stock that demonstrated typical nursing behaviour (inserting their heads into cells containing larvae) were retrieved from the colonies. The nurse bees were placed into 7.0×3.5×3.5 cm plastic tubes containing a moist paper towel and brought to the laboratory (<5 min transit time). There, bees were incubated at 4°C until movement was reduced. Next, they were mounted onto individual plastic holders, and affixed with removable tape behind the head and across the thorax (Supporting Figure S1A). After restraining, the bees were fed 2 µl of 30% sucrose solution before being starved for 2 h at 37°C, 65–70% RH.

### Quantification of gustatory responsiveness

After the 2 h starvation period, gustatory responsiveness [54], [55] was measured by the proboscis extension response (PER). Bees were observed for PER while being stimulated with H_2_O, followed by six sucrose solutions (sucrose in H_2_O) in an ascending order (0.1%, 0.3%, 1%, 3%, 10%, 30%) at a minimum interval of 2 min between trials. Thereafter, bees were assigned a gustatory response score (GRS) that totalled the number of times PER was observed throughout the seven trials. The maximum GRS of 7 indicated that bees responded to all sucrose concentrations and H_2_O (high gustatory responsiveness). In contrast, bees with a GRS of 0 did not respond to any of the seven stimuli. GRS provides a measure for the subjective value that the bee places on sucrose solutions, which are later used as rewards in the associative learning paradigm (see below). Thus, via GRS quantification, we ensured that only bees that responded to a reward (and thus could be rewarded) were trained [42], [56].

### Quantification of associative learning ability

Because we used 30% sucrose solution as reward [42], [56], only bees that showed a PER response to a solution of at least 30% sucrose were allowed to participate in the associative learning assay. Over the course of the study, 48 bees did not respond to 30% sucrose and were thus not trained. As olfactory stimuli [35], 2 µl of each of two odours (carnation and cineole) were applied to separate pieces of filter paper, which were then placed into two different 10 ml syringes (BD Luer-Lock™ Tip). Each bee was initially stimulated for 6 sec directly to the antennae with approximately 6 ml of the carnation odour, which served as the conditioned stimulus (CS) during associative conditioning (see below). Thereafter, the alternative odour (cineole) was administered in the same manner. Bees that responded spontaneously to either odour were omitted (N = 57), as we could not validate learning for individuals whose response to the CS was spontaneous prior to conditioning [42].

During conditioning, each bee was subjected to six CS reward pairings with an approximate inter-trial interval of 15 min. During every trial, bees were stimulated with 6 ml of carnation odour applied directly to their antennae for 6 sec. Using a Gilmont® syringe, the final 3 ml of the CS was paired with 1 µl of 30% sucrose reward for 3 sec in order to form an association between the two [42]. For each trial, those bees who displayed PER to the odour stimulus prior to the introduction of the reward were recorded as positive, while the bees that did not respond prior to reward were noted as negative for PER.

Following the six conditioning trials, we performed a retention test where the specific CS memory of the worker bees was evaluated. The bees were first presented with cineole (the alternative odour), and then CS without reward. The outcome was not associated with survival time in hyperoxia: Longevity was the same whether bees demonstrated specific CS memory (did not respond to alternative odour, N = 304) or not (responded to alternative odour, N = 86, Student t-test; t = −0.022, P = 0.983, Supporting Figure S2).

Bees that responded to the final CS without reward were given a learning score ranging from 1 to 5, reflecting the total number of conditioning trials in which PER was observed minus the number of responses to the alternative odour (this number was 0 for 304 bees and 1 for 86 bees, above). The learning score, thereby, took into account how precise the learning was. Bees that did not respond to the final CS without reward and had not responded to any of the prior six conditioning trials were given a learning score of 0. Finally, the few bees that responded in all or some conditioning trials but did not respond to the final CS presentation without reward were omitted (N = 14), as we could not validate the learned association in them (details in [42]).

For all trials, bees were placed in front of a neutral air stream approximately 8 sec before and after odour stimulation. A minimum of 5 min passed between trials to prevent habituation effects [42]. The general activity of each bee was also monitored in every trial to ensure that all animals remained healthy.

### Survival in hyperoxia

Bees that completed the olfactory conditioning test and received a measure of learning ability (learning scores 0–5) were placed in hyperoxia to monitor survival capability. Hyperoxia induces features of premature senescence in many laboratory systems, and can provide a reproducible test of metabolic stress resistance that often, but not without exception [39], [57], is relevant to lifespan in a general way [36]–[38]. Bees were housed in 1.5 ml Eppendorf tubes that had two holes on top and an opening at the bottom for animal waste (Supporting Fig. S1B). The experimental bees were kept in an incubator (HERAcell O_2_/CO_2_, Thermo Scientific) with a constant 80% O_2_ concentration; incubator temperature was 34°C and RH averaged 63±2%. A standard diet of 1.5 g of ground pollen per 30 ml of 30% sucrose solution was administered twice per day into a pipette tip that rested in one of the holes atop the Eppendorf tube (Supporting Fig. S1B). The other hole was left unobstructed for breathing.

Survivorship censuses took place four times daily: 7–8 AM; 1–2 PM; 6–7 PM; 11 PM–12 AM until the last bee was observed dead. As needed, alive bees were transferred to fresh tubes in order to prevent bacterial and/or fungal growth. Individuals that were likely harmed during routine transfers were excluded. Individual lifespans were calculated as the number of hours spent in hyperoxia prior to observed death.

### Statistics

The datasets on associative learning, gustatory responsiveness and survival time in hyperoxia conformed to Levene's and Bartlett's tests of equal variance and parametric statistics were used [58]. The relationships between learning ability, gustatory responsiveness, and survival were tested with Pearson's correlations, and investigated further with the Proportional hazard (Cox) regression, which we have applied to bee survival data in previous studies [23], [59]. One-sided analysis of covariance (ANCOVA) for comparison of regression curve slopes was used to test differences between olfactory learning [58]. Log-linear transformation was tested but the outcome was similar to raw data (for bees surviving ≤50 h vs. >50 h in hyperoxia: F = 3.79, P = 0.046; for bees not responding vs. responding to sucrose at ≤0.1% in H_2_O: F = 21.18, P = 0.002). Thus, results from the untransformed dataset were reported. To control for variance linked to social environment (different single-cohort colonies) and age at testing (18–22 vs. 32–36 days old), we utilized Main effect ANOVA (MANOVA) with learning ability, colony, and age-group as categorical predictors of survival. This reporting was preferred over the Cox regression with colony or age-group as stratifying variables, because the Cox regression model does not allow the input of three predictors. Yet, the significant effect of learning performance on survival capability persisted even in the stratified Cox regression analyzes in which colony or sampling age were controlled for separately (χ^2^ = 4.678, P = 0.031; χ^2^ = 4.738, P = 0.030, respectively, N = 341). The relationship between the binary outcome of the retention test (bees demonstrating specific CS memory, or not) and the survival time in hyperoxia was tested with a Student t-test using survival time as the dependent variable. All analyses performed in Statistica 6.0 (StatSoft).

## Supporting Information

Figure S1 (A) Worker honeybee prepared for testing of Pavlovian learning ability. The restraint holder is custom-made from Plexiglas, and the bee was affixed with straps of tape. After quantification of gustatory responsiveness and learning, the straps were removed and the bee was released unharmed. (B) Worker bee in the modified Eppendorf tube design used in our assay of survival capability in hyperoxia. The lid has holes for feeding and air-exchange. The end of the tube is cut open and sealed with cotton to absorb animal waste.
(10.12 MB TIF)

Figure S2 Relationship between the outcome of the retention test, where bees were monitored for specific CS memory, and survival time in hyperoxia (80% O2). Bars are averages + S.E. for survival time (hours). Specific CS memory for the conditioned stimulus (carnation) was measured after olfactory conditioning by presenting the bees with an alternative odour (cineole) one time. No (zero) proboscis extension response (PER) to the alternative odour demonstrated specific CS memory. The performance in the retention test was not associated with survival time in hyperoxia (Student t-test; t = −0.022, P = 0.983). Sample sizes inside bars.
(0.01 MB TIF)
