# Supplementary figures and images for: Honeybee Associative Learning Performance and Metabolic Stress Resilience Are Positively Associated
Source: PLoS One. 2010 Mar 17;5(3):e9740. doi: 10.1371/journal.pone.0009740 (PMC2840029; doi:10.1371/journal.pone.0009740)

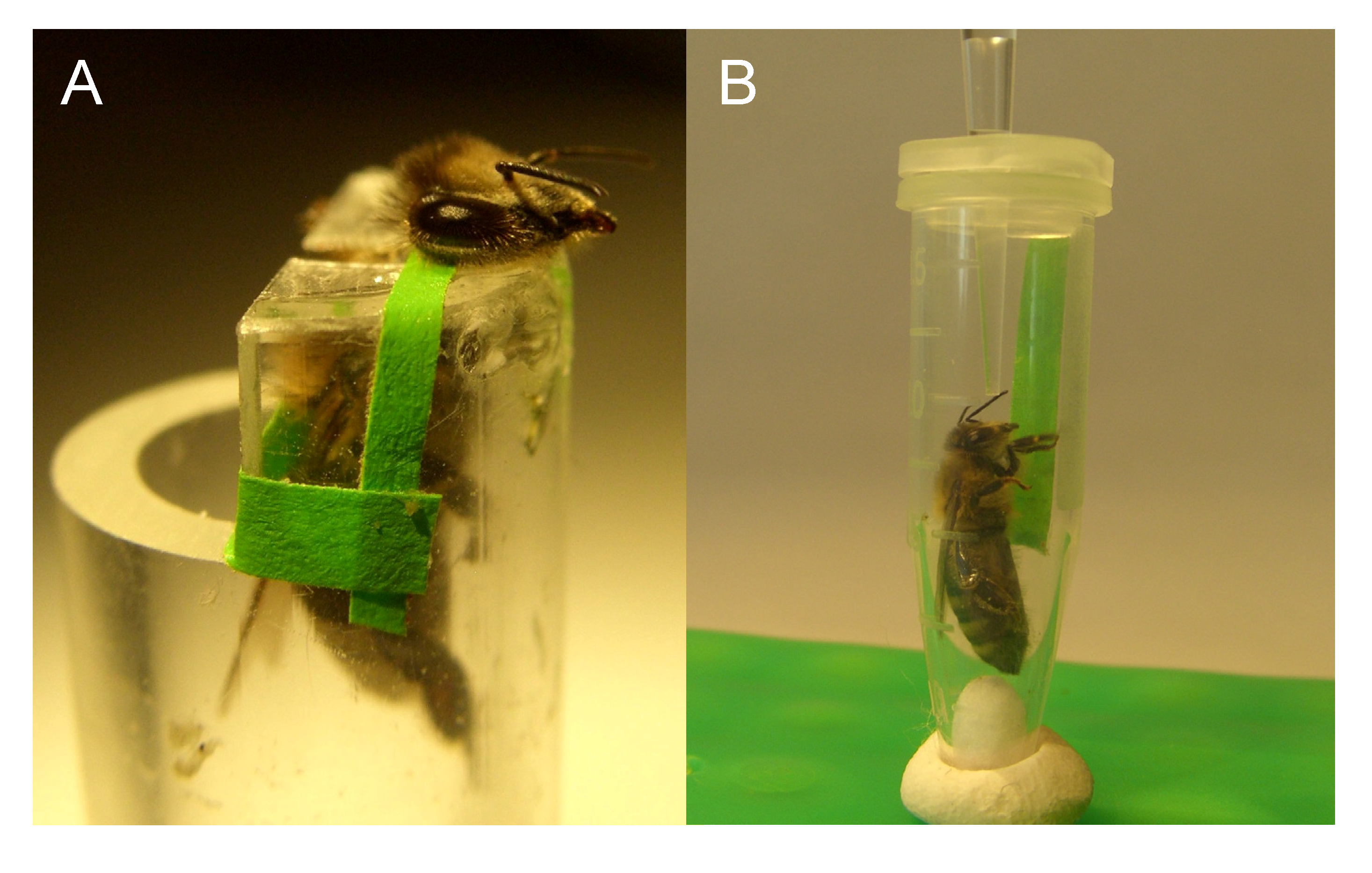

Supplement: Figure S1 — (A) Worker honeybee prepared for testing of Pavlovian learning ability. The restraint holder is custom-made from Plexiglas, and the bee was affixed with straps of tape. After quantification of gustatory responsiveness and learning, the straps were removed and the bee was released unharmed. (B) Worker bee in the modified Eppendorf tube design used in our assay of survival capability in hyperoxia. The lid has holes for feeding and air-exchange. The end of the tube is cut open and sealed with cotton to absorb animal waste. (10.12 MB TIF) [file pone.0009740.s001.tif]
